# Apoptosis control and proliferation marker in human normal and neoplastic adrenocortical tissues

**DOI:** 10.1038/sj.bjc.6600287

**Published:** 2002-05-03

**Authors:** G P Bernini, A Moretti, P Viacava, A G Bonadio, P Iacconi, P Miccoli, A Salvetti

**Affiliations:** Department of Internal Medicine, University of Pisa, Via Roma 67, 56100 Pisa, Italy; Department of Oncology, University of Pisa, Via Roma 67, 56100 Pisa, Italy; Department of Surgery, University of Pisa, Via Roma 67, 56100 Pisa, Italy

**Keywords:** P53, Bcl-2, Ki-67, adrenal cortex, adrenocortical tumours

## Abstract

We evaluated by immunohistochemistry the expression of the Bcl-2 and p53 proteins, as markers of apoptosis control, and of MIB-1, as a marker of cell proliferation, in a series of normal and neoplastic adrenocortical tissues. The specimens were 13 normal adrenals, 13 aldosterone-producing adenomas, 13 non-functioning adenomas and 16 carcinomas. Results were calculated as percentage of immunostained cells by using specific antibodies. No p53 protein was detected in any of the adrenocortical adenomas (functioning and non functioning) or normal adrenals, while p53 was overexpressed in 15 out of 16 carcinomas. In particular, 10 adrenal cancer specimens (62.5%) showed strong staining in a high percentage (range 10–50%) of the malignant cells. The percentage of Bcl-2 positive cells was higher (*P*<0.05 or less) in non-functioning adenomas (8.1±1.9%) and in carcinomas (14.9±5.6%) than in normals (2.9±0.9%) and aldosterone-producing adenomas (5.3±1.3%) since four specimens of the non-functioning adenomas-group (30.7%) and six of the carcinomas-group (37.5%) showed over 10% positivity (cut-off for normal values, set at 90th percentile of our controls). MIB-1 positivity was 0.50±0.36% in normals, 0.54±0.08% in non-functioning adenomas and 0.54±0.08% in aldosterone-producing adenomas. MIB-1 was expressed in all carcinomas with values (13.7±3.1%) significantly (*P*<0.0006) higher than in the other groups. In conclusion, the present data indicate that the apoptosis control and proliferation activity evaluated by the p53 and MIB-1 proteins are impaired in adrenal carcinomas but preserved in adenomas, independently of their functional status. Therefore, these immunohistochemical markers, overexpressed in carcinomas only, may be useful in the diagnosis of malignancy in adrenocortical tumours. Whether Bcl-2 positivity found in some carcinomas and non-functioning adenomas may constitute, in the latter, a negative prognostic marker is still unknown.

*British Journal of Cancer* (2002) **86**, 1561–1565. DOI: 10.1038/sj/bjc/6600287 www.bjcancer.com

© 2002 Cancer Research UK

## 

The growth and diffusion of cancers, and consequently their aggressiveness, are dependent on a complicated balance between several mechanisms, such as neoangiogenesis, release of growth stimulating and inhibiting factors, apoptosis control and proliferation activity (Fox, 1997).

While malignant tumours of human adrenocortical glands are very rare (Wooten and King, 1993), benign adenomas, both non-functioning and functioning, are common in clinical practice. However, the apoptosis control and proliferation activity of neoplasms of the adrenal cortex have not been adequately investigated and results obtained to date are not univocal.

Mutations in the p53 tumour suppressor gene, which constitute the most common type of genetic alteration in several human cancers (Velculescu and El-Deiry, 1996), do not seem to be frequent in adrenocortical tumours (Ohgaki *et al*, 1993; Reincke *et al*, 1994; Reincke *et al*, 1996). Different results were found in studies on pathological adrenal cortex (Lin *et al*, 1994; Reincke *et al*, 1994, 1996; Barzon *et al*, 2001) for what concerns p53 overexpression, a well-established immunohistochemical marker of p53 mutations (Davidoff *et al*, 1991),

Bcl-2, a cellular oncogene which encodes for proteins that inhibit apoptosis, thereby determining resistance to programmed cell death (Hockenbery *et al*, 1990, 1993), is overexpressed in different types of solid tumours (Pilotti *et al*, 1994; Vargas *et al*, 1997). However, Bcl-2 immunoreactivity has been demonstrated not only in adrenal cortical lesions (cortical hyperplasia, adenomas and carcinomas) but also in normal cortical tissue (Fogt *et al*, 1998).

MIB-1, which recognises a nuclear antigen (Ki-67) associated with all phases of the cell cycle except in resting cells (Gerdes *et al*, 1984), is overexpressed in several human tumours and is commonly considered very useful in the evaluation of tumour cell growth (Hall and Woods, 1990). Recently it has also been reported that MIB-1 expression differentiates benign from malignant adrenocortical tumours but not adenomas from normal cortex (Iino *et al*, 1997; Nakazumi *et al*, 1998).

The aim of our study was to evaluate, by immunohistochemistry, expression of the Bcl-2 and p53 proteins as markers of apoptosis control and of MIB-1 protein as a marker of cell proliferation, in a series of human normal and neoplastic (adenomas and carcinomas) adrenocortical tissues.

## MATERIALS AND METHODS

### Patients and pathology

The study, approved by the local Ethical Committee, involved 55 patients with aldosterone-producing adenomas (*n*=13, APA), non-functioning cortical adenomas (*n*=13, NFA) and adrenocortical carcinomas (*n*=16, CA). As controls, 13 normal adrenal glands (N) were studied after removal from patients with renal carcinomas. Clinical and pathological details of our population are shown in Table 1

**Table 1:**
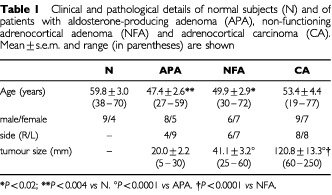
Clinical and pathological details of normal subjects (N) and of patients with aldosterone-producing adenoma (APA), non-functioning adrenocortical adenoma (NFA) and adrenocortical carcinoma (CA). Mean±s.e.m. and range (in parentheses) are shown

.

Patients with APA showed the typical pattern of the disease with hypertension (systolic blood pressure 174±7.0 mmHg, mean±s.e., diastolic blood pressure 107±3.5 mmHg) hypokaliemia (2.9±0.2 mmol l^−1^), low PRA levels (0.18±0.06 ng ml h^−1^) and high plasma aldosterone (1298±172 pmol l^−1^). In patients with NFA adrenal masses had been incidentally discovered and endocrinological investigation had revealed normal catecholamines, glucocorticoids, androgens and mineralcorticoids in all cases. Patients with CA had been investigated for abdominal pain in 11 cases and for hyperandrogenism in five cases. Pathological diagnosis of our tumours was based on accepted criteria including tumour mass, presence of metastasis or recurrence, mitotic ratio, nuclear grade, necrosis and capsula and/or vascular invasion (Lack, 1997). In particular, tumour necrosis was absent in two, low in two, moderate in four and wide in eight cases. Staging of CA, according to the Mac-Farlane criteria (Mac-Farlane, 1958), indicated stage II in six cases, stage III in eight cases and stage IV in two patients.

After surgery, all NFA and APA were cured while 13 CA died of local and/or diffuse metastases with a mean(±s.d.) survival of 27.2±31.0 months (range 3–100 months). Three patients were alive at time of writing of the manuscript (follow-up performed 10 months after surgery).

### Specimens

A total of 55 formalin-fixed, paraffin-embedded blocks of adrenal tissues were studied. Five μm sections were stained with haematoxilin-eosin for histological evaluation. Five additional μm sections were used for immunohistochemistry.

### Immunohistochemistry

#### Antibodies

The sections were incubated with the following primary monoclonal antibodies: MAb 124 (DBA, dilution 1 : 100) for Bcl-2, MAb DO7 (Novocastra Laboratories, dilution 1 : 100) for p53, and MIB-1 (DBA, dilution 1 : 200) for Ki-67. Incubation time was 12–24 h at 4°C.

### Method

Tissue sections stained for MIB-1 were pretreated using 0.1 trypsin with 0.1 calcium chloride buffered to pH 7.6. In order to unmask the antigens, the slides were microwave treated in 10 mM citrate buffer, pH6, for a total of 10 min. MIB-1 and p53 sections were incubated with biotin-labelled secondary antibody (dilution 1 : 500) and avidin-biotin complex (Vector Burlingame, CA, USA) for 30 min respectively. 3-3′ diaminobenzidine tetrahydrochloride with 0.01 hydrogen peroxide was used as chromogen. The alkaline phosphatase-anti-alkaline phosphatase (APAAP) method was used to amplify the Bcl-2 signal. The reaction was developed with alkaline phosphatase containing naphthol, AS-MX, fast red, and levamisol (APAAP kits DAKO, Milan, Italy).

### Controls

Positive controls consisted of invasive breast carcinoma known to express MIB-1 and p53 antigens. Positive control for Bcl-2 was follicular non-Hodgkin lymphoma. Negative controls were obtained by omitting primary antibodies.

### Evaluation of parameters

The positivity index was obtained by counting almost 500 cells of lesions of normal tissue on three ×250 fields and calculating the percentage of cells with nuclear (p53 and MIB-1) and cytoplasmatic (Bcl-2) immunoreactivity. Since in our normal tissues no immunoreactivity was found for p53 and MIB-1, the specimens were considered positive when at least 1% of cells showed distinct nuclear staining. However, in order to reduce possible false positive results, we also analysed our data by moving the cut-off of normality to 5%. In contrast, for Bcl-2 the specimens were considered positive when at least 11% of cells showed intense cytoplasmatic immunoreactivity, as the cut-off of normal values, calculated as 90th percentile of our controls, proved to be 10%. All parameters were determined independently by two pathologists (VP and BA) and discordant cases were solved by simultaneous review of the specimens. Cases with interobserver differences greater than 5% were re-evaluated.

### Statistics

All data are expressed as mean±s.e.m. Values obtained for each variable to compare groups were analysed by using the unpaired *t*-test and Wilcoxon's test. Spearman correlation coefficients (*r*) were used as parameters of association. Statistical difference was accepted at *P*<0.05.

## RESULTS

Age in the APA and NFA-groups was significantly lower than in N and, among adrenal lesions, tumour size in CA was highest while that in APA proved to be the lowest (Table 1).

**Figure 1:**
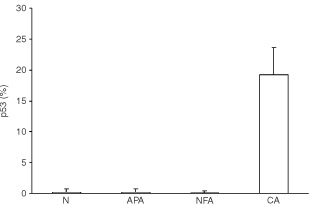
Mean percentage (±s.e.m.) of p53 positive cells in normal cortex (N), Aldosterone-producing adenomas (APA), non-functioning adenomas (NFA) and carcinomas (CA).

No p53 protein was detected in any sample of N, APA and NFA. In contrast, p53 was expressed in 15 out of 16 (94%) CA and in 10 specimens the staining involved between 10–50% of the malignant cells (Figures 1

and 2

**Figure 2:**
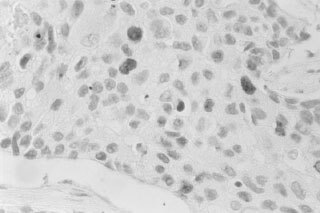
Adrenocortical carcinoma with several p53 immunoreactive cells.

). Even considering our cut-off of positivity ⩾5%, p53 expression was still elevated, corresponding to 11 out of 16 cases (68.7%).

**Figure 3:**
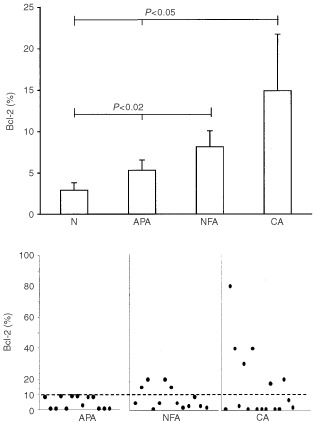
Mean percentage (±s.e.m.) of Bcl-2 positive cells in normal cortex (N), Aldosterone-producing adenomas (APA), non-functioning adenomas (NFA) and carcinomas (CA). The statistical difference is shown (above). Bcl-2 individual data in APA, NFA and CA: the dotted line represents the upper limit of normal calculated as 90th percentile of our controls (below).

APA (5.3±1.3%) and N (2.9±0.9%) -groups showed an average percentage of Bcl-2 positive cells lower than NFA (8.1±1.9%) and CA (14.9±5.6%)-groups. No difference was found between NFA and CA (Figure 3

). Analytical data showed that four specimens of the NFA-group (30.7%) and six of the CA-group (37.5%) had positivity over 10% (our cut-off of normal values) (Figure 3 below).

**Figure 4:**
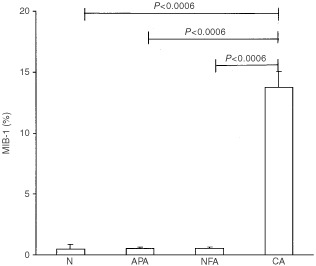
Mean percentage (±s.e.m.) of MIB-1 positive cells in normal cortex (N), Aldosterone-producing adenomas (APA), non-functioning adenomas (NFA) and carcinomas (CA). The statistical difference is shown.

MIB-1 expression was very low (⩽1%) in cortical adenomas (APA and NFA) and in normal specimens, while strong immunoreactivity (3–40%) was detected in all CA with mean values (13.7±3.0%) significantly (*P*<0.0001) higher than in normal adrenals (0.5%), APA (0.53±0.08%) and NFA (0.53±0.08%) (Figures 4

and 5

**Figure 5:**
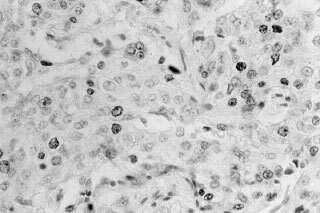
Adrenocortical carcinoma with immunoreactive MIB-1 cells.

).

No correlation was found between p53, MIB-1 and Bcl-2 or between these immunohistochemical markers and clinical or pathological variables in the APA and NFA-groups. Positive associations between p53 and MIB-1 (*r*=0.560, *P*<0.02) and between p53 and tumour necrosis (*r*=0.530, *P*<0.03) were detected in CA.

## DISCUSSION

The aim of our study was to investigate apoptosis control and proliferation activity in a group of human adrenal neoplasms, using an immunohistochemical approach. This method presents some intrinsic limits, both because it does not always reflect a corresponding genetic alteration and because it does not permit exact quantification of the proteins in the tissues. However, immunohistochemistry sometimes appears to be the only procedure able to detect specific proteins involved in tumorogenesis and offers the advantage of detecting the exact intratissutal localisation and the intracellular distribution of several markers.

It is well-known that the human tumour suppressor p53 is deemed critical for maintaining genomic stability and homeostasis (Agarwal *et al*, 1998). Accordingly, mutations in the p53 gene constitute the most common type of genetic alteration in human cancers (Hollstein *et al*, 1991; Velculescu and El-Deiry, 1996). However, few data refer to adrenocortical CA and those reported indicate that p53 mutations are uncommon in these tumours (Ohgaki *et al*, 1993; Reincke *et al*, 1994). Different findings were observed by immunohistochemistry. Our study shows frequent p53 immunoreactivity in CA and other authors (Reincke *et al*, 1994) reported p53 positivity in about half of their cases. The definition of positive immunohistochemical staining in this study was ⩾1% because no immunostaining was detected in the specimens of our controls. However, even when the cut-off of p53 positivity was moved to 5% in order to reduce possible false positive results, we obtained similar results with high frequency of immunoreactivity in CA (68.7%). Since genetic analysis of the specimens could not be performed, we cannot establish whether the p53 overexpression we found represents underlying gene mutations or results from abnormal stabilisation of the wild-type protein leading to an increase in its half-life. In any case, our finding indicates that p53 immunoreactivity is a feature of CA. Different results have been reported in APA where no or poor p53 immunoreactivity was found not only in adenomas without p53 mutations (Reincke *et al*, 1996) but also in those with ascertained genetic alterations (Lin *et al*, 1994; Adleff *et al*, 1998). The present data confirm these observations since immunohistochemistry failed to show p53 positive cells in all 13 APA. All these findings imply that in APA p53 mutations are likewise uncommon but do not exclude the possibility that in these adenomas the immunohistochemical method may have low sensitivity. Indeed a mutant p53 protein may be not recognisable by the antibody, deletion mutations do not necessarily induce abnormal p53 protein and in the case of missense mutations several events may be required for p53 overexpression (Casey *et al*, 1996). More homogeneous data on the p53 protein have been observed in NFA. No genetic p53 mutation and no p53 immunoreactivity was detected in isolated cases (Reincke *et al*, 1994) and in a group of NFA (Adleff *et al*, 1998). Our results fit with these data since none of the NFA patients studied was positive for p53 immunoreactivity.

Taken together, the present results indicate that p53 is a useful immunohistochemical marker to distinguish benign from malignant adrenal tumours since it proved to be overexpressed in several carcinomas and never expressed in functioning and non-functioning adenomas.

Bcl-2 is a cellular oncogene which encodes for proteins that inhibit apoptosis, thus determining resistance to programmed cell death (Hockenbery *et al*, 1990, 1993). Bcl-2 expression has been reported in normal tissues but also in different types of solid tumours (Pilotti *et al*, 1994; Vargas *et al*, 1997). However few studies have investigated this protein in adrenal glands. The most extensive study (Fogt *et al*, 1998) indicated that Bcl-2 is overexpressed in normal adrenal cortex, without distinct staining difference between normal and pathological tissue (hyperplasia, adenomas and carcinomas). Our results confirm that Bcl-2 positivity is detectable in normal adrenals. In contrast, cortical adenomas and carcinomas showed heterogeneous Bcl-2 expression. Some CA (37.5%) and NFA (30.7%) had strong positivity, while in the remaining and in all APA the number of Bcl-2 positive cells was similar to normal tissue. The prognostic significance of this particular immunohistochemical pattern observed in some NFA is unknown.

Evaluating our data, we observed a strong discrepancy in the expression rate of Bcl-2 and p53 in CA. We have no clear explanation for this finding, although it may depend on the fact that the two markers involve different and independent mechanisms of apoptosis control. Thus, in human tumours the expression of Bcl-2 and mutant p53 does not always coexist, even if both markers block programmed cell death. Accordingly, a lack of relation and even an inverse association between Bcl-2 and p53 were found in breast carcinomas (Leek *et al*, 1994; Siziopiku *et al*, 1996; Viacava *et al*, 1999) and in parathyroid adenomas (Naccarato *et al*, 1998).

A useful tool to examine cell proliferation activity consists in immunohistochemical analysis by MIB-1, an antibody that recognises Ki-67, a cell cycle-related nuclear antigen (Gerdes *et al*, 1984; Hall and Woods, 1990). A recent study reported no difference in MIB-1 expression between normal cortex and functioning (Iino *et al*, 1997; Nakazumi *et al*, 1998) and non-functioning cortical adenomas (Gerdes *et al*, 1984). In contrast, MIB-1 may differentiate adenomas from carcinomas of the adrenal cortex and may predict the biological behaviour of adrenocortical CA (Suzuki *et al*, 1992; Goldblum *et al*, 1993; Sasano *et al*, 1995; Iino *et al*, 1997; Nakazumi *et al*, 1998). Our results confirm these data, showing that in cortical adenomas, independently of their function, MIB-1 expression is similar to that in normal cortex, while in malignant tumours MIB-1 positivity appears to be strongly and constantly present.

In conclusion, the present data indicate that the apoptosis control and proliferation activity evaluated by the p53 and MIB-1 proteins are impaired in CA but preserved in adenomas, independently of their functional status. Therefore, these immunohistochemical markers, overexpressed in CA only, may be useful in the diagnosis of malignancy in adrenocortical tumours. Finally, our results show that Bcl-2 overexpression may occur in CA but also in some NFA. Whether Bcl-2 positivity constitutes a negative prognostic marker in NFA is, at present, unknown.
